# The impact of ovulation-suppressing contraceptives on behavioral and functional difficulties in borderline personality disorder

**DOI:** 10.1038/s41386-024-02045-4

**Published:** 2025-01-11

**Authors:** Seyma Katrinli, Alex O. Rothbaum, Raneeka DeMoss, William C. Turner, Ben Hunter, Abigail Powers, Vasiliki Michopoulos, Alicia K. Smith

**Affiliations:** 1 Department of Gynecology and Obstetrics, Emory University, Atlanta, USA; 2 Skyland Trail, Atlanta, USA; 3 Department of Psychiatry & Behavioral Sciences, Emory University, Atlanta, USA

**Keywords:** Outcomes research, Psychiatric disorders

## Abstract

Borderline Personality Disorder (BPD) is characterized by rapidly shifting emotional, interpersonal, and behavioral symptoms, often co-morbid with mood and anxiety disorders. Females are more likely to be diagnosed with BPD than males and exhibit greater functional impairment. Hormonal fluctuations may influence the manifestation of BPD symptoms. Here, we investigated the influence of ovulation-suppressing contraceptives on behavioral and functional difficulties in BPD. The sample included 348 females ages 18-50 undergoing residential treatment for psychiatric disorders, with 131 having a BPD diagnosis. Patients were categorized by their contraceptive method: Ovulation-suppressing contraceptives (N = 145) and naturally cycling (N = 203). Interaction models tested the impact of ovulation-suppressing contraceptives on the relationship between BPD diagnosis and behavioral and functional difficulties at admission and discharge, assessed by the four Behavior and Symptom Identification Scale (BASIS-32) domains: difficulties in relationships, daily living, depression/anxiety, and impulsivity. Females with a BPD diagnosis were more likely to use ovulation-suppressing contraceptives compared to those without BPD (p = 0.04). However, ovulation-suppressing contraceptive use was not associated with behavioral and functional difficulties at admission, discharge, or over time. Ovulation-suppressing contraceptives moderated the association between BPD diagnosis and difficulties in relationships (p = 0.004), difficulties in daily living (p = 0.01), and depression/anxiety symptoms (p = 0.004). Specifically, patients with BPD experienced more behavioral and functional difficulties only if naturally cycling, whereas patients without BPD showed higher symptom severity only if using ovulation-suppressing contraceptives. Our findings suggest that the impact of ovulation-suppressing contraceptives on behavioral and functional difficulties varies depending on BPD diagnosis and underscores the need for further clinical studies.

## Introduction

Borderline personality disorder (BPD) is a severe psychiatric condition that affects 6% of the adult population. The prevalence of BPD reaches up to 10% in psychiatric outpatients and 20% in psychiatric inpatients [1]. Individuals with BPD suffer from rapidly shifting emotional, interpersonal, and behavioral symptoms, including unstable and chaotic interpersonal relationships, intense and highly variable emotions, impulsive behavior, aggressiveness, and chronic suicidality and self-harm behaviors [2]. Due to the rapid shifts in symptoms, BPD is difficult to diagnose, and its symptoms are challenging to manage [2]. Indeed, BPD is highly co-morbid with other psychiatric disorders, including major depressive disorder (MDD) and post-traumatic stress disorder (PTSD), with over 70% of individuals with BPD meeting the criteria for other psychiatric diagnoses [3].

BPD is predominantly diagnosed in females and displays distinct differences in symptoms and co-morbidities between sexes [4]. Males with BPD are more likely to exhibit aggressiveness and impulsivity, often co-morbid with substance use disorders. In contrast, females tend to show emotional instability, suicidal or self-harm behaviors, and unstable relationships, along with comorbid anxiety and mood disorders [4]. The biological mechanism behind these rapid shifts in symptoms may be linked to ovarian hormones, given their fluctuations across a female’s lifespan—puberty, pregnancy, menopause—and even within the menstrual cycle. Indeed, growing evidence suggests that fluctuations in ovarian hormones play a significant part in the increased prevalence of MDD, PTSD, and BPD, in females [2, 5–10]. Specifically, within-person variations but not absolute levels of estrogen correlated with higher BPD symptoms [11]. Moreover, prior research has shown that individuals with higher BPD traits tend to experience increased symptoms and negative interpersonal emotions (i.e., anxiety, rejection sensitivity, irritability, and aggression) during phases of the menstrual cycle characterized by lower estrogen and higher progesterone levels, such as the mid-luteal and perimenstrual phases [8–10]. Given the impact of fluctuating estrogen and progesterone levels on mental health symptoms, contraceptives that suppress ovulation and stabilize estrogen and progesterone fluctuations (i.e., ovulation-suppressing contraceptives) may impact the manifestation and severity of symptoms related to BPD. However, research examining whether ovulation-suppressing contraceptives improve or worsen mental health symptoms is conflicting [12–16]. Rather than a straightforward association, the recent evidence suggests a complex relationship between ovulation-suppressing contraceptives and mental health outcomes depending on psychiatric history [14]. Specifically, while hormonal contraceptives were associated with decreased depressive symptoms in individuals with a history of psychiatric disorders, in individuals without a history of psychiatric disorders, hormonal contraceptives increased the risk of depression [14]. Additionally, recent studies have highlighted the complexity of hormonal contraceptive effects on psychiatric symptoms, which vary depending on the type of progestin used (e.g., drospirenone, norethindrone), the dose of ethinylestradiol, and dosing schedules (e.g., 21-7, 24-4, or continuous) [12, 17, 18]. Non-progesterone-receptor-mediated effects may also contribute to the diverse psychiatric outcomes observed in females using hormonal contraceptives. For example, while population-based studies using large national registries have shown increased risks for internalizing symptoms such as depression and anxiety in females using hormonal contraceptives [19], clinical trials have demonstrated the benefits of specific contraceptives, such as those containing drospirenone, in managing premenstrual dysphoric disorder (PMDD) [20]. These studies suggest that the effects of hormonal contraceptives are not uniform and may depend on individual sensitivities to different formulations and schedules. Thus, while hormonal contraceptives may exacerbate symptoms in some individuals, they could offer therapeutic benefits for others, particularly in the context of specific psychiatric conditions such as PMDD.

However, prior studies predominantly focused on symptoms of depression and anxiety, which may have different underlying mechanisms than BPD [12–15]. Despite multiple studies establishing the link between fluctuating ovarian hormones and BPD symptoms [11, 12, 14], only one small study to date examined the role of oral contraceptive pills, but not other hormonal contraceptives, in BPD symptoms [13]. In this study of 17 females, the use of oral contraceptive pills worsened BPD symptoms [13]. To delineate the broader impact of ovulation-suppressing contraceptives in BPD, we sought to investigate how ovulation-suppressing contraceptives influence behavioral and functional difficulties in females with and without a BPD diagnosis.

## Methods

### Participants

The participants were patients who were admitted to a non-profit residential and ambulatory psychiatric rehabilitation facility in the southeastern United States. We conducted a medical review of 413 non-menopausal and non-pregnant patients with female sex assigned at birth, ages 18–50, who received residential treatment between 01/01/2019 and 02/14/2024. All participants have a primary diagnosis of a mood disorder, anxiety disorder, psychotic disorder, or neurodevelopmental disorder. The study was approved by the Institutional Review Board of Emory University. All participants consented to the use of their clinical data in research activities as part of their consent for treatment, and the complete waiver of HIPAA authorization and informed consent has been granted by the IRB.

### Measures

All demographic, clinical, and medical information, including psychiatric diagnoses, assessments, and contraception methods, were abstracted from medical records from the intake visit and initial history and physical. BPD diagnosis was determined using the DSM-5 criteria [1] during clinical interviews with the admitting psychiatrists in the psychiatric treatment program. Behavioral and functional difficulties were assessed at admission and discharge by the four subscales of the 32-item Behavior and Symptom Identification Scale (BASIS-32), which are difficulties in relationships, difficulties in daily living, depression and anxiety, and impulsivity [21]. The BASIS-32 measures the severity and impact of various symptoms and functional problems associated with psychiatric disorders [21].

Contraception methods were abstracted from patients’ current medications in their medical records. Eligible participants were on contraception at the time of admission to the residential treatment, and they stayed on contraception during their treatment. All medication, including contraceptives, are distributed by nursing staff to residential patients with visible confirmation of ingestion if oral. Females were divided into two groups based on their contraceptive method. The ovulation-suppressing contraceptive group included the contraceptives that suppress ovulation, which are oral contraceptive pills (OCP), depot medroxyprogesterone acetate (DMPA) injections, contraceptive patches, contraceptive implants, and vaginal rings. The majority of participants in the ovulation-suppressing contraceptive group (74%) were on combination OCPs, while 20% using a progestin-only contraceptive method such as DMPA, implant, or progestin-only pill. The naturally cycling group included females who were not using any contraceptives. For the current study, 65 females using IUDs were excluded, as IUDs may suppress ovulation in some individuals but not others [22]. The final sample included 348 females with 131(37.6%) having a BPD diagnosis and 145 (41.7%) using ovulation-suppressing contraceptives.

### Statistical analysis

We performed multiple linear regressions to test the association of behavioral and functional difficulties at admission and discharge with co-morbid BPD diagnosis and ovulation-suppressing contraceptive use. In addition, we conducted linear mixed models with a random intercept for participants to evaluate the association of behavioral and functional difficulties in the overall sample that combines admission and discharge measures. To explore these associations longitudinally, we used linear mixed models with behavioral and functional difficulties at admission and discharge as the dependent variable, co-morbid BPD diagnosis or ovulation-suppressing contraceptives as the main effect, (co-morbid BPD diagnosis x time point) or (ovulation-suppressing contraceptives x time point) as the interaction term, and a random intercept for subjects.

Finally, we examined ovulation-suppressing contraceptive use as the moderator of the associations between behavioral and functional difficulties at admission and discharge (dependent variable) and co-morbid BPD diagnosis (independent variable). To further explore these relationships, we conducted stratified analyzes. First, we stratified by ovulation-suppressing contraceptive use, performing separate analyzes for patients using ovulation-suppressing contraceptives and those who were naturally cycling to test the associations between BPD diagnosis (independent variable) and behavioral and functional difficulties (dependent variable) in each group. Second, we stratified by BPD diagnosis, performing separate analyzes for patients with and without a BPD diagnosis to test the associations between ovulation-suppressing contraceptive use (independent variable) and behavioral and functional difficulties in each subgroup (dependent variable). All models included age as a covariate. We also examined the moderation effect longitudinally, by incorporating a three-way interaction term (co-morbid BPD diagnosis x ovulation-suppressing contraceptives x time point) in the models. All analyzes were performed using R Statistical Software (v4.2.1; R Core Team 2022).

## Results

### Demographic and clinical characteristics

Demographic and clinical characteristics of the analytical sample are presented in Table 1 and Supplementary Table 1. Participants with a co-morbid BPD diagnosis are more likely to be younger, have a longer length of inpatient stay, and have a primary diagnosis of a mood disorder (e.g., bipolar disorder, major depressive disorder; Table 1). Females using ovulation-suppressing contraceptives are also more likely to be younger than naturally cycling females and have a longer duration of inpatient stay (Supplementary Table 1).

**Table 1:** Demographic and clinical characteristics.

Mean (SD) or *N* (%)	Co-morbid BPD (*N* = 131)	No BPD diagnosis (*N* = 217)	*p*-value
Age	23.19 (5.27)	26.87 (8.02)	4.4e-7
Race^a^			0.97
White	104 (79.4%)	177 (81.6%)	
Black	10 (7.6%)	18 (8.3%)	
Asian	6 (4.6%)	8 (3.7%)	
Other	7 (5.3%)	11 (5.1%)	
Primary Diagnosis^b^			5.8e-5
Mood disorder	124 (94.7%)	183 (84.33%)	
Anxiety disorder	4 (3.1%)	6 (2.8%)	
Psychotic disorder	0 (0%)	24 (11.1%)	
Personality disorder	3 (2.3%)	3 (1.4%)	
Ovulation-suppressing contraceptive use	64 (48.9%)	81 (37.3%)	0.043
Length of stay	53.7 (18.62)	47.51 (22.99)	0.014

^a^Race was missing for 7 participants. ^b^Mood disorders include bipolar disorder, major depressive disorder, and persistent depressive disorder. Anxiety disorders include generalized anxiety disorder. Psychotic disorders include schizophrenia, schizoaffective disorder, delusional disorder, and psychosis. Personality disorders include BPD for co-morbid BPD cases and other personality disorders (e.g., narcissistic, avoidant) for patients without a BPD diagnosis. P-values are from t-tests for continuous variables and Fisher’s exact tests for categorical variables.

### Associations between co-morbid BPD and behavioral and functional difficulties

We first examined the difference in the severity of behavioral and functional difficulties between females with and without a co-morbid BPD diagnosis. Females with a BPD diagnosis exhibited more difficulties in relationships and impulsive behaviors in the overall sample, as well as at admission and discharge separately (Table 2, Supplementary Fig. 1A,D). Additionally, females with a co-morbid BPD diagnosis showed a greater improvement in impulsivity compared to those who do not have BPD (β[SE] = −0.28[0.08], *p* = 6.1e-4, Supplementary Fig. 1D, Supplementary Table 2). However, this greater improvement associated with BPD diagnosis might be due to patients with BPD having higher impulsive behaviors to begin with (Supplementary Fig. 1D). Patients with co-morbid BPD compared to those without BPD exhibited more severe depression and anxiety symptoms only at discharge (Table 2, Supplementary Fig. 1C). Finally, BPD was associated with more difficulties in daily living in the overall sample but not when examined separately at admission and discharge (Table 2). The associations were largely consistent with the same direction of effect in the sensitivity analyzes adjusting for primary diagnosis (Supplementary Table 3).

**Table 2:** The associations between co-morbid BPD and behavioral and functional difficulties.

Admission (*N* = 348 samples)			
Psychiatric symptoms	Beta (β)	SE	*p*-value
Difficulties in relationships	0.27	0.11	**0.012**
Difficulties in daily living	0.21	0.11	0.060
Depression and Anxiety	0.20	0.11	0.060
Impulsivity	0.37	0.08	**9.0e-6**

Discharge (*N* = 348 samples)			
Psychiatric Symptoms	Beta (β)	SE	*p*-value
Difficulties in relationships	0.24	0.09	**0.010**
Difficulties in daily living	0.15	0.10	0.126
Depression and Anxiety	0.23	0.10	**0.025**
Impulsivity	0.13	0.05	**0.017**

Longitudinal analysis (*N* = 696 samples)			
Psychiatric Symptoms	Beta (β)	SE	*p*-value
Difficulties in relationships	0.28	0.10	**0.005**
Difficulties in daily living	0.21	0.10	**0.038**
Depression and Anxiety	0.20	0.10	0.059
Impulsivity	0.39	0.07	**1.9e-8**

The models include psychiatric symptoms as the dependent variable, co-morbid BPD as the independent variable, and age as a covariate. The longitudinal model includes data from admission and discharge, and the associations were tested using linear mixed models with a random intercept for participants. Significant associations were shown in bold. SE: Standard error.

### Ovulation-suppressing contraceptive use and behavioral and functional difficulties

We next evaluated the association of ovulation-suppressing contraceptive use with co-morbid BPD diagnosis behavioral and functional difficulties. Females with a BPD diagnosis are more likely to use ovulation-suppressing contraceptives (OR[95% CI] = 1.60[1.01, 2.55], *p* = 0.043) compared to females who do not have BPD, but the association did not remain significant after adjusting for age and primary diagnosis (β[SE] = 0.37[0.24], *p* = 0.12). Ovulation-suppressing contraceptive use was not associated with behavioral and functional difficulties at admission or discharge, and there was no change in symptom severity pre- to post-treatment (Supplementary Table 4).

### The impact of ovulation-suppressing contraceptives on behavioral and functional difficulties in BPD

**Fig. 1:**
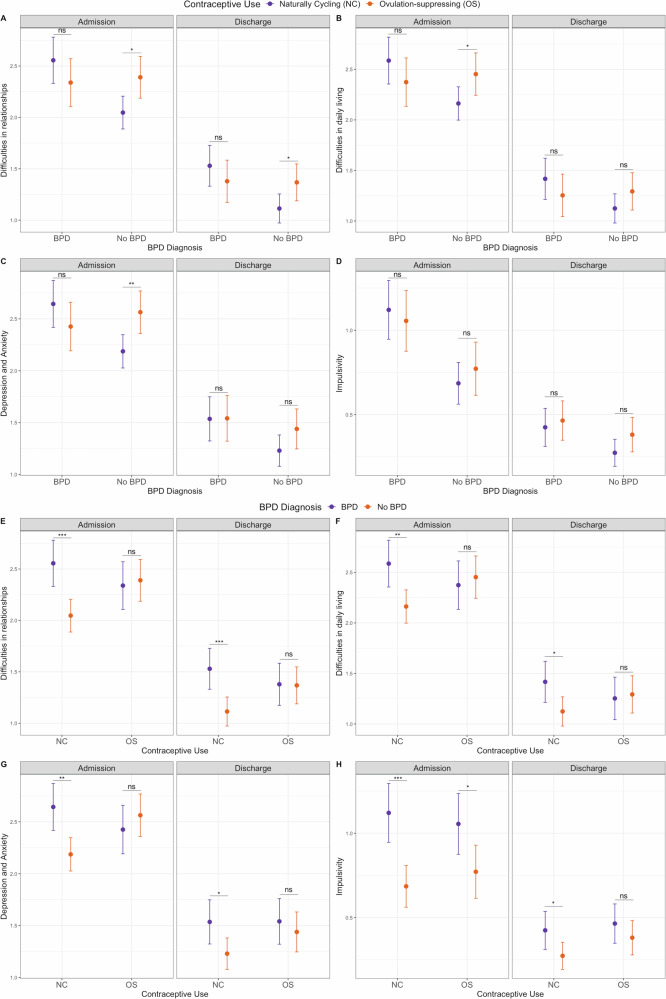
Interactions between BPD diagnosis and ovulation-suppressing contraceptive use on behavioral and functional difficulties. The associations between ovulation-suppressing contraceptive use and **A** Difficulties in relationships, **B** Difficulties in daily living, **C** Depression and anxiety symptoms, and **D** Impulsivity in females with and without BPD diagnosis at admission and discharge. The associations between BPD diagnosis and **E** Difficulties in relationships, **F** Difficulties in daily living, **G** Depression and anxiety symptoms, and **H** Impulsivity in ovulation-suppressing contraceptive users (OS) and naturally cycling (NC) females at admission and discharge. The y-axis shows estimated marginal means and standard errors for psychiatric symptoms. Statistical significance is indicated by asterisks (*p < 0.05, **p < 0.01, ***p < 0.001). Significant interactions between ovulation-suppressing contraceptives and BPD diagnosis were observed for difficulties in relationships at admission and discharge **A**,**E**, difficulties in daily living at admission **B**,**F**, and depression and anxiety symptoms **C**,**G**. ns: Not significant (*p* > 0.05).

Finally, we investigated the influence of ovulation-suppressing contraceptives on the association between BPD diagnosis and behavioral and functional difficulties. Ovulation-suppressing contraceptive use moderated the association between BPD and difficulties in relationships both at admission and discharge, and in the overall sample (Table 3, Supplementary Fig. 2), but did not influence the amelioration of the other symptoms in those with and without a BPD diagnosis (Supplementary Tables 5 and 6). Patients with a BPD diagnosis compared to those without BPD reported more difficulties in relationships at admission and discharge, as well as in the overall sample, only if they are naturally cycling (i.e., not using ovulation-suppressing contraceptives, Table 4, Fig. 1E). When stratified by BPD diagnosis, the use of ovulation-suppressing contraceptives was associated with more difficulties in relationships at admission and discharge, as well as in the overall sample, only in patients without BPD (Table 4, Fig. 1A).

**Table 3:** Ovulation-suppressing contraceptives as the moderator of the associations between co-morbid BPD and behavioral and functional difficulties.

Admission (*N* = 348 samples)			
Psychiatric Symptoms	Interaction Beta (β)	Interaction SE	Interaction *p*-value
Difficulties in relationships	−0.56	0.21	**0.008**
Difficulties in daily living	−0.50	0.21	**0.020**
Depression and Anxiety	−0.59	0.21	**0.005**
Impulsivity	−0.15	0.16	0.35

Discharge (N = 348 samples)			
Psychiatric Symptoms	Interaction Beta (β)	Interaction SE	Interaction *p*-value
Difficulties in relationships	−0.41	0.18	**0.029**
Difficulties in daily living	−0.33	0.19	0.080
Depression and Anxiety	−0.20	0.20	0.30
Impulsivity	−0.07	0.10	0.52

Longitudinal analysis (N = 696 samples)			
Psychiatric Symptoms	Interaction Beta (β)	Interaction SE	Interaction *p*-value
Difficulties in relationships	−0.56	0.20	**0.004**
Difficulties in daily living	−0.50	0.20	**0.013**
Depression and Anxiety	−0.59	0.20	**0.004**
Impulsivity	−0.16	0.14	0.25

The models include psychiatric symptoms as the dependent variable, co-morbid BPD as the independent variable, ovulation-suppressing contraceptives x BPD as the interaction term, and age as a covariate. The longitudinal model includes data from admission and discharge, and the associations were tested using linear mixed models with a random intercept for participants. The statistics were for the interaction effects (Ovulation-suppressing contraceptives x BPD). Significant associations were shown in bold. SE: Standard error.

**Table 4:** The associations of behavioral and functional difficulties with BPD diagnosis, stratified by ovulation-suppressing contraceptive use, and with contraceptive use, stratified by BPD diagnosis.

Admission	Ovulation-suppressing contraceptive use (Independent Variable)						BPD Diagnosis (Independent Variable)					
	With BPD diagnosis (*N* = 131)			Without BPD diagnosis (*N* = 217)			Ovulation-suppressing contraceptive users (*N* = 145)			Naturally Cycling (*N* = 203)		
Psychiatric Symptoms (Dependent Variable)	β	SE	*p*-value	β	SE	*p*-value	β	SE	*p*-value	β	SE	*p*-value
Difficulties in relationships	−0.22	0.15	0.15	0.35	0.14	**0.012**	−0.05	0.14	0.75	0.51	0.15	**0.001**
Difficulties in daily living	−0.22	0.16	0.17	0.29	0.14	**0.038**	−0.06	0.15	0.69	0.41	0.16	**0.009**
Depression and Anxiety	−0.23	0.15	0.14	0.38	0.14	**0.006**	−0.15	0.15	0.32	0.47	0.15	**0.002**
Impulsivity	−0.07	0.13	0.58	0.09	0.10	0.37	0.26	0.12	**0.035**	0.45	0.11	**7.6e-5**

Discharge												
Psychiatric Symptoms (Dependent Variable)	β	SE	*p*-value	β	SE	*p*-value	β	SE	*p*-value	β	SE	*p*-value
Difficulties in relationships	−0.14	0.15	0.33	0.25	0.12	**0.032**	0.02	0.14	0.88	0.41	0.13	**0.001**
Difficulties in daily living	−0.16	0.15	0.30	0.16	0.12	0.17	−0.03	0.15	0.83	0.29	0.13	**0.027**
Depression and Anxiety	0.006	0.16	0.97	0.21	0.12	0.08	0.09	0.16	0.61	0.32	0.13	**0.013**
Impulsivity	0.04	0.08	0.57	0.11	0.07	0.12	0.08	0.09	0.35	0.15	0.07	**0.024**

Longitudinal												
Psychiatric Symptoms (Dependent Variable)	β	SE	*p*-value	β	SE	*p*-value	β	SE	*p*-value	β	SE	*p*-value
Difficulties in relationships	−0.21	0.15	0.16	0.35	0.13	**0.006**	−0.04	0.14	0.80	0.51	0.14	**2.4e-4**
Difficulties in daily living	−0.21	0.15	0.17	0.29	0.13	**0.025**	−0.06	0.15	0.67	0.42	0.14	**0.003**
Depression and Anxiety	−0.23	0.16	0.15	0.37	0.13	**0.004**	−0.16	0.16	0.31	0.46	0.14	**8.8e-4**
Impulsivity	−0.06	0.11	0.59	0.10	0.08	0.25	0.29	0.11	**0.007**	0.47	0.09	**5.2e-7**

The models include psychiatric symptoms as the dependent variable, ovulation-suppressing contraceptive use (1st and 2nd columns) or co-morbid BPD (3rd and 4th columns) as the independent variable, and age as a covariate. The longitudinal model includes data from admission and discharge, and the associations were tested using linear mixed models with a random intercept for participants. Significant associations were shown in bold. SE: Standard error.

Ovulation-suppressing contraceptives moderated the association between BPD and difficulties in daily living and the severity of depression and anxiety symptoms in the overall sample, as well as at admission, but not at discharge (Table 3, Supplementary Fig. 2). The severity of the symptoms was higher in patients with BPD who are naturally cycling compared to naturally cycling females without BPD (Table 4, Fig. 1F, G). Similar to the difficulties in relationships, ovulation-suppressing contraceptives did not influence the reduction of either the severity of depression and anxiety symptoms or difficulties in daily living in patients with and without co-morbid BPD (Supplementary Tables 5 and 6). When stratified by BPD diagnosis, the use of ovulation-suppressing contraceptives was associated with higher depression and anxiety symptoms and more difficulties in daily living at admission and in the overall sample, only in patients without BPD (Table 4, Fig. 1B, C). The associations remained largely consistent, showing similar effects in the sensitivity analyzes that adjusted for primary diagnosis (Supplementary Tables 7 and 8).

The association between a co-morbid BPD diagnosis and higher impulsivity was consistent in patients using ovulation-suppressing contraceptives and naturally cycling patients (Table 4, Fig. 1H), and this association was not moderated by ovulation-suppressing contraceptives (Table 3, Supplementary Fig. 2). When stratified by ovulation-suppressing contraceptive use, the improvement in impulsivity associated with BPD was only observed in naturally cycling females (Supplementary Table 6, Supplementary Fig. 3) and not moderated by the use of ovulation-suppressing contraceptives (Supplementary Table 5). The lack of a moderating effect from ovulation-suppressing contraceptives suggests that it does not alter the fundamental association between BPD and the reduction of impulsive behaviors, but it may influence the extent of this reduction, making it more pronounced in naturally cycling females. The full regression model outputs for the interaction models (Table 3) are shown in Supplementary Table 9 and the full regression model outputs for the stratified analyzes (Table 4) are shown in Supplementary Tables 10 and 11.

## Discussion

Females with BPD experience severe and rapidly shifting emotional, interpersonal, and behavioral symptoms, often co-morbid with mood and anxiety disorders [1, 3]. Recent evidence highlights the role of fluctuating ovarian hormones in these rapid shifts and the severity of symptoms related to BPD. [2, 8–11] Here, we investigated the impact of ovulation-suppressing contraceptives on behavioral and functional difficulties in those with and without a BPD diagnosis to explore whether contraceptives that suppress ovulation, and with it the fluctuations in estrogen and progesterone, may impact the manifestation and severity of psychiatric outcomes related to BPD.

In the current study, the use of ovulation-suppressing contraceptives was not associated with the severity of behavioral and functional difficulties either at admission and discharge or change in symptom severity pre- to post-treatment. Although females with a co-morbid BPD diagnosis were more likely to use ovulation-suppressing contraceptives compared to females without, the association did not remain significant after adjusting for age and primary diagnosis. This finding is consistent with an earlier study reporting higher levels of BPD symptoms in females using oral contraceptives [11]. However, we cannot assess the directionality of this association with our current sample, since the participants who were on ovulation-suppressing contraceptives had them prescribed before initiating the residential treatment and we do not have any information as to whether they were prescribed ovulation-suppressing contraceptives to manage their behavioral symptoms or for other reasons (i.e., birth control, menstrual regulation).

The reports regarding whether hormonal contraceptives increase or decrease the risk of mental health disorders are mixed [12–16]. However, recent evidence suggests that the association between hormonal contraceptives and mental health outcomes may depend on individuals’ psychiatric history [14]. While some studies reported mood-related disturbances as a side effect of contraceptives in the general population [12, 13], oral contraceptive pills were shown to be effective in the treatment of PMDD and premenstrual worsening of depressive symptoms in females with MDD [23–26]. Moreover, a recent meta-analysis reported that the use of hormonal contraceptives was associated with a decrease in depressive symptoms in females with pre-existing psychiatric disorders but an increased risk of depression in females without any history of psychiatric disorders [14]. Therefore, in addition to examining the associations between BPD, ovulation-suppressing contraceptives, and psychiatric outcomes, we sought to explore the interaction between ovulation-suppressing contraceptives and BPD. As expected, a co-morbid BPD diagnosis was associated with more severe behavioral and functional difficulties, specifically increased difficulties in relationships, impulsivity, depression, anxiety symptoms, and difficulties in daily living. Importantly, we showed that ovulation-suppressing contraceptives moderated the association of BPD with difficulties in relationships, depression, and anxiety symptoms, and difficulties in daily living, such that the associations between BPD and the severity of psychiatric outcomes are more pronounced in naturally cycling females, compared to females using ovulation-suppressing contraceptives. We also observed a differential association between ovulation-suppressing contraceptives and difficulties in relationships, depression and anxiety symptoms, and daily living, depending on BPD diagnosis. Specifically, the use of ovulation-suppressing contraceptives was associated with more severe symptoms, but only in individuals without co-morbid BPD. The use of ovulation-suppressing contraceptives did not influence the associations between BPD and changes in behavioral and functional difficulties during treatment. These findings suggest that higher behavioral and functional difficulties associated with the use of ovulation-suppressing contraceptives may be less pronounced in individuals with BPD, potentially due to ovulation-suppressing contraceptives helping to regulate the rapidly shifting emotional, interpersonal, and behavioral symptoms characteristic of BPD. This aligns with recent evidence showing that the use of hormonal contraceptives is associated with decreased psychiatric symptoms among females with pre-existing mental disorders, but an increased risk of psychiatric symptoms in females without prior mental health conditions [14]. The mechanisms behind this differential impact of ovulation-suppressing contraceptives on behavioral and functional difficulties between those with and without BPD are yet to be elucidated. One potential mechanism may be through stabilizing estrogen and progesterone fluctuations. Ovulation-suppressing contraceptives could reduce emotional reactivity by preventing estrogen and progesterone fluctuations [27] or, specifically, the prevention of allopregnanolone—a metabolite of progesterone—flux during the luteal phase, which has been linked to irritability and interpersonal difficulties [9, 10]. Studies have shown that preventing the metabolism of progesterone to allopregnanolone can alleviate PMDD symptoms, suggesting that stabilizing these hormonal pathways may mitigate the emotional and behavioral symptoms seen in BPD. [20, 28–30] Alternatively, ovulation-suppressing contraceptives reduce testosterone levels which were shown to be elevated in those with BPD [31–33]. In addition to hormonal mechanisms, social determinants of health (e.g., socioeconomic status (SES), access to healthcare) can also contribute to observed differences in behavioral and functional difficulties between patients with BPD using ovulation-suppressing contraceptives and those who do not. Individuals prescribed ovulation-suppressing contraceptives may have better access to healthcare, including gynecological and psychiatric care, contributing to earlier detection and management of psychiatric symptoms.

Finally, our findings align with the hypothesis that BPD may involve a sensitivity to hormonal fluctuations similar to that observed in PMDD. Specifically, the observed moderating effect of ovulation-suppressing contraceptives on the association between BPD and difficulties may be attributed to the stabilization of progesterone and its metabolite allopregnanolone. The Dimensional Affective Sensitivity to Hormones across the Menstrual Cycle (DASH-MC) framework proposes that individuals with heightened sensitivity to hormonal flux, particularly surges in allopregnanolone, experience pronounced affective changes, such as irritability and interpersonal difficulties, during the luteal phase [34]. This transdiagnostic sensitivity to hormone flux may contribute to similar patterns of emotional reactivity in individuals with BPD, suggesting that the benefits of ovulation-suppressing contraceptives could be related to preventing these hormonal surges.

Although the study has numerous strengths, including a comprehensive investigation of behavioral and functional difficulties longitudinally, it is not without limitations. First, the duration of contraceptive use varied among participants, which could confound our analyzes. Second, we did not perform subgroup analyzes on different types of ovulation-suppressing contraceptives (i.e., combined oral contraceptives, progestin-only methods, etc.) due to power considerations. Third, we do not have information on the temporal history of ovulation-suppressing contraceptive use (i.e., whether patients were prescribed contraceptives before or after the manifestation of their psychiatric symptoms). It is also possible that patients who are naturally cycling may have discontinued hormonal contraceptives due to adverse mood effects, which are often associated with increased sensitivity to hormonal fluctuations across the menstrual cycle. This would suggest that the natural cycling group could represent a more hormone-sensitive subgroup, potentially leading to a stronger association between hormonal fluctuations and mood disturbances in this group compared to those on contraceptives. The younger age of patients on contraceptives, as observed in our sample, may align with this interpretation, as younger individuals may be less likely to have experienced adverse effects from contraceptives or may not have used them long enough to discontinue due to mood-related side effects. Thus, we cannot determine causality or directionality—whether BPD comorbidity predicts the severity of symptoms in those using ovulation-suppressing contraceptives and are naturally cycling, or whether ovulation-suppressing contraceptive use predicts symptom levels in individuals with and without BPD. Finally, we did not account for the menstrual cycle phase and its interaction with contraceptives, as we lacked ovarian hormone measures and menstrual cycle length information to accurately phase participants’ cycles. While hormone levels in those using ovulation-suppressing contraceptives would not provide much insight, the phase of the menstrual cycle could still influence psychiatric symptoms in naturally cycling individuals [15]. Yet, our longitudinal analyzes included measures at two time points, which to some degree account for within-subject variability due to the menstrual cycle phase.

Overall, our findings suggest that ovulation-suppressing contraceptives may help to regulate the rapidly shifting emotional, interpersonal, and behavioral symptoms in those with BPD by stabilizing estrogen and progesterone fluctuations. This opens up new avenues for treatment and highlights the need for further research to confirm these results and explore additional therapeutic options.

## Supplementary information

Supplemental Material

Supplementary Tables 9-11

## Data Availability

The datasets generated and/or analyzed during the current project are not publicly available due to sensitive information contained in patient medical records. De-identified data are available from the corresponding author (seyma.katrinli@emory.edu) upon reasonable request.
