# Supplementary material for: The impact of ovulation-suppressing contraceptives on behavioral and functional difficulties in borderline personality disorder
Source: Neuropsychopharmacology. 2025 Jan 11;50(5):841–8. doi: 10.1038/s41386-024-02045-4 (PMC11914153; doi:10.1038/s41386-024-02045-4)
Supplement: Supplementary file 1 — Supplemental Material [file 41386_2024_2045_MOESM1_ESM.pdf]

Supplementary Table 1: Demographic and clinical characteristics described by Ovulation-suppressing birth control use

Mean (SD) or N (%)	Ovulation-suppressing contraceptive users (N = 145)	Naturally Cycling (N = 203)	p-value
Age	24.32 (6.63)	26.31 (7.69)	0.01
Race[a]			0.45
White	119 (82.1%)	162 (79.8%)	
Black	8 (5.5%)	20 (9.9%)	
Asian	5 (3.4%)	9 (4.4%)	
Other	9 (6.2%)	9 (4.4%)	
Primary Diagnosis[b]			0.30
Mood disorder	133 (91.7%)	174 (85.7%)	
Anxiety disorder	2 (1.4%)	8 (3.9%)	
Psychotic disorder	7 (4.8%)	17 (8.4%)	
Personality disorder	3 (2.1%)	3 (8.4%)	
Length of stay	24.32 (6.63)	26.31 (7.69)	0.01

[a]Race was missing for 7 participants. [b]Mood disorders include bipolar disorder, major depressive disorder, and persistent depressive disorder. Anxiety disorders include generalized anxiety disorder. Psychotic disorders include schizophrenia, schizoaffective disorder, delusional disorder, and psychosis. Personality disorders include BPD for co-morbid BPD cases and other personality disorders (e.g., narcissistic, avoidant) for patients without a BPD diagnosis. P-values are from t-tests for continuous variables and Fisher's exact tests for categorical variables.

Supplementary Table 2: The associations between co-morbid BPD and change in behavioral and functional difficulties

Psychiatric Symptoms	Beta (β)	SE	p-value
Difficulties in relationships	-0.04	0.12	0.704
Difficulties in daily living	-0.07	0.11	0.54
Depression and Anxiety	0.04	0.11	0.74
Impulsivity	-0.28	0.08	**6.1e-4**

The statistics were for the interaction term (BPD x Time Point). Significant associations were shown in bold. SE: Standard error.

Supplementary Table 3: Sensitivity analyses for the associations between co-morbid BPD and behavioral and functional difficulties

Admission (N = 348 samples)			
Psychiatric Symptoms	Beta (β)	SE	p-value
Difficulties in relationships	0.19	0.11	0.082
Difficulties in daily living	0.13	0.11	0.24
Depression and Anxiety	0.12	0.11	0.28
Impulsivity	0.39	0.08	**6.85e-6**
Discharge (N = 348 samples)			
Psychiatric Symptoms	Beta (β)	SE	p-value
Difficulties in relationships	0.22	0.10	**0.025**
Difficulties in daily living	0.12	0.10	0.228
Depression and Anxiety	0.18	0.10	0.074
Impulsivity	0.14	0.05	**0.012**
Longitudinal Analysis* (N = 696 samples)			
Psychiatric Symptoms	Beta (β)	SE	p-value
Difficulties in relationships	0.23	0.16	**0.025**
Difficulties in daily living	0.16	0.10	0.12
Depression and Anxiety	0.13	0.10	0.21
Impulsivity	0.40	0.07	**1.16e-8**

* Longitudinal analysis includes data from admission and discharge, and the associations were tested using linear mixed models with a random intercept for participants.
The models were adjusted for age and primary diagnosis. Significant associations were shown in bold. SE: Standard error.

Supplementary Table 4: The associations between Ovulation-suppressing contraceptive use and behavioral and functional difficulties

Admission			
Psychiatric Symptoms	Beta (β)	SE	p-value
Difficulties in relationships	0.14	0.10	0.17
Difficulties in daily living	0.11	0.11	0.31
Depression and Anxiety	0.16	0.10	0.13
Impulsivity	0.06	0.08	0.49
Discharge			
Psychiatric Symptoms	Beta (β)	SE	p-value
Difficulties in relationships	0.11	0.09	0.21
Difficulties in daily living	0.05	0.09	0.60
Depression and Anxiety	0.15	0.10	0.13
Impulsivity	0.09	0.05	0.08
Longitudinal Analysis*			
Psychiatric Symptoms	Beta (β)	SE	p-value
Difficulties in relationships	-0.04	0.12	0.73
Difficulties in daily living	-0.07	0.11	0.56
Depression and Anxiety	-0.03	0.11	0.98
Impulsivity	0.004	0.08	0.97

* Longitudinal analysis includes data from admission and discharge, and the associations were tested using linear mixed models with a random intercept for participants and the statistics are for the interaction term (Ovulation-suppressing contraceptives x Time Point). The models were adjusted for age. Significant associations were shown in bold. SE: Standard error.

Supplementary Table 5: Ovulation-suppressing contraceptives as the moderator of the associations between co-morbid BPD and change in behavioral and functional difficulties

Psychiatric Symptoms	Beta (β)	SE	p-value
Difficulties in relationships	0.16	0.24	0.51
Difficulties in daily living	0.17	0.23	0.46
Depression and Anxiety	0.39	0.23	0.09
Impulsivity	0.09	0.17	0.58

The statistics were for the 3-way interaction term (Ovulation-suppressing contraceptives x BPD x Time Point). The models were adjusted for age. SE: Standard error.

Supplementary Table 6: The associations between co-morbid BPD and change in behavioral and functional difficulties in Ovulation-suppressing contraceptive users and naturally cycling females

	Ovulation-suppressing contraceptive users (N = 145)			Naturally Cycling (N = 203)		
Psychiatric Symptoms	Beta (β)	SE	p-value	Beta (β)	SE	p-value
Difficulties in relationships	0.05	0.16	0.77	-0.11	0.17	0.51
Difficulties in daily living	0.03	0.17	0.85	-0.14	0.16	0.37
Depression and Anxiety	0.25	0.18	0.16	-0.13	0.15	0.36
Impulsivity	-0.24	0.13	0.07	-0.33	0.11	**0.002**

The statistics were for the interaction term (BPD x Time Point). The models were adjusted for age. Significant associations were shown in bold. SE: Standard error.

Supplementary Table 7: Sensitivity analyses for interaction models testing the moderating effect of Ovulation-suppressing contraceptives

Admission (N = 348 samples)			
Psychiatric Symptoms	Beta (β)	SE	p-value
Difficulties in relationships	-0.51	0.21	**0.015**
Difficulties in daily living	-0.47	0.22	**0.031**
Depression and Anxiety	-0.56	0.21	**0.008**
Impulsivity	-0.15	0.16	0.349
Discharge (N = 348 samples)			
Psychiatric Symptoms	Beta (β)	SE	p-value
Difficulties in relationships	-0.38	0.19	**0.041**
Difficulties in daily living	-0.32	0.19	0.092
Depression and Anxiety	-0.18	0.20	0.356
Impulsivity	-0.10	0.11	0.348
Longitudinal Analysis* (N = 696 samples)			
Psychiatric Symptoms	Beta (β)	SE	p-value
Difficulties in relationships	-0.53	0.20	**0.008**
Difficulties in daily living	-0.48	0.20	**0.018**
Depression and Anxiety	-0.57	0.20	**0.006**
Impulsivity	-0.17	0.14	0.209

*Longitudinal analysis includes data from admission and discharge, and the associations were tested using linear mixed models with a random intercept for participants.
The statistics were for the interaction term (Ovulation-suppressing contraceptives x BPD). The models were adjusted for age and primary diagnosis. Significant associations were shown in bold. SE: Standard error.

Supplementary Table 8: Sensitivity analysis for the associations between co-morbid BPD and behavioral and functional difficulties in Ovulation-suppressing contraceptive users and naturally cycling females

	Ovulation-suppressing contraceptive users (N = 145)			Naturally Cycling (N = 203)		
Admission						
Psychiatric Symptoms	Beta (β)	SE	p-value	Beta (β)	SE	p-value
Difficulties in relationships	-0.10	0.15	0.489	0.41	0.16	**0.011**
Difficulties in daily living	-0.13	0.15	0.418	0.32	0.16	0.051
Depression and Anxiety	-0.22	0.16	0.162	0.37	0.15	**0.017**
Impulsivity	0.25	0.13	**0.047**	0.44	0.12	**2.2e-4**
Discharge						
Psychiatric Symptoms	Beta (β)	SE	p-value	Beta (β)	SE	p-value
Difficulties in relationships	-0.007	0.15	0.960	0.35	0.13	**0.007**
Difficulties in daily living	-0.06	0.15	0.706	0.25	0.13	0.066
Depression and Anxiety	0.04	0.17	0.799	0.26	0.13	0.052
Impulsivity	0.07	0.09	0.437	0.17	0.07	**0.018**
Longitudinal Analysis*						
Psychiatric Symptoms	Beta (β)	SE	p-value	Beta (β)	SE	p-value
Difficulties in relationships	-0.08	0.15	0.587	0.43	0.14	**0.002**
Difficulties in daily living	-0.11	0.15	0.472	0.35	0.14	**0.016**
Depression and Anxiety	-0.22	0.16	0.179	0.38	0.14	**0.007**
Impulsivity	0.28	0.11	**0.009**	0.47	0.09	**1.1e-6**

*Longitudinal analysis includes data from admission and discharge, and the associations were tested using linear mixed models with a random intercept for participants.

The models were adjusted for age and primary diagnosis. Significant associations were shown in bold. SE: Standard error.

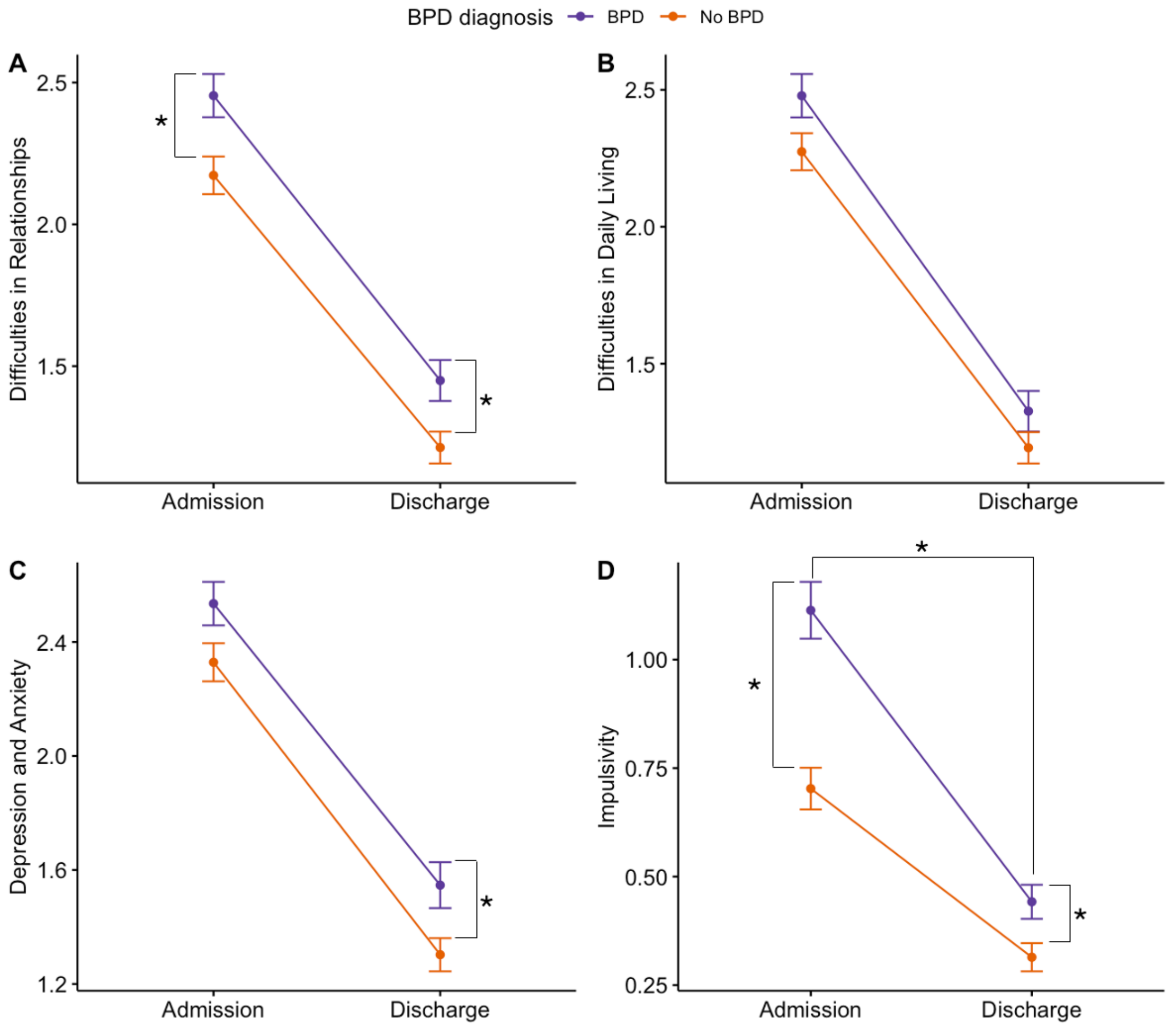

Supplementary Figure 1: Associations between co-morbid BPD and **A)** Difficulties in relationships, **B)** Difficulties in daily living, **C)** Depression and anxiety symptoms, and **D)** Impulsivity. Significant differences between BPD and no-BPD groups in behavioral and functional difficulties at admission and discharge, and reduction of behavioral and functional difficulties were indicated by asterisk (*). BPD diagnosis was associated with increased difficulties in relationships at admission and discharge (A), increased depression symptoms at discharge (C), increased impulsivity at admission and discharge (D), and greater reduction of impulsivity pre- to post-treatment (D).

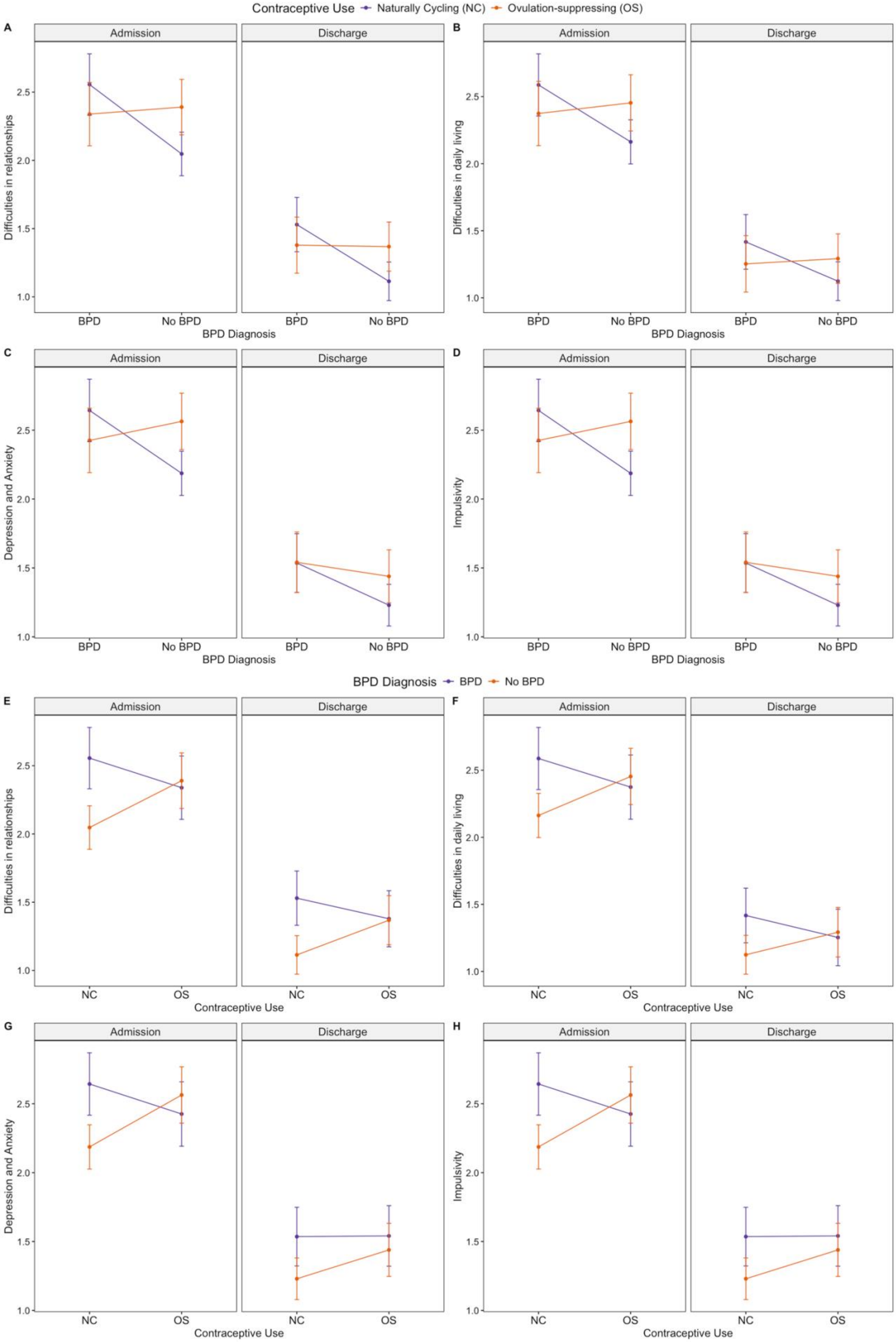
Contraceptive Use
Naturally Cycling (NC)
Ovulation-suppressing (OS)
A
B
C
D
Admission
Discharge
Difficulties in relationships
Difficulties in daily living
Depression and Anxiety
Impulsivity
BPD
No BPD
BPD Diagnosis
BPD Diagnosis
BPD
No BPD
E
F
G
H
NC
OS
Contraceptive Use

Supplementary Figure 2: The associations between ovulation-suppressing contraceptive use and **A)** Difficulties in relationships, **B)** Difficulties in daily living, **C)** Depression and anxiety symptoms, and **D)** Impulsivity in females with and without BPD diagnosis at admission and discharge. The associations between BPD diagnosis and **E)** Difficulties in relationships, **F)** Difficulties in daily living, **G)** Depression and anxiety symptoms, and **H)** Impulsivity in ovulation-suppressing contraceptive users (OS) and naturally cycling (NC) females at admission and discharge. The y-axis shows estimated marginal means and standard errors for psychiatric symptoms. Significant interactions between ovulation-suppressing contraceptives and BPD diagnosis were observed for difficulties in relationships at admission and discharge (A and E), difficulties in daily living at admission (B and F), and depression and anxiety symptoms (C and G). Significant interactions between ovulation-suppressing contraceptives and BPD diagnosis were indicated by an asterisk (*).

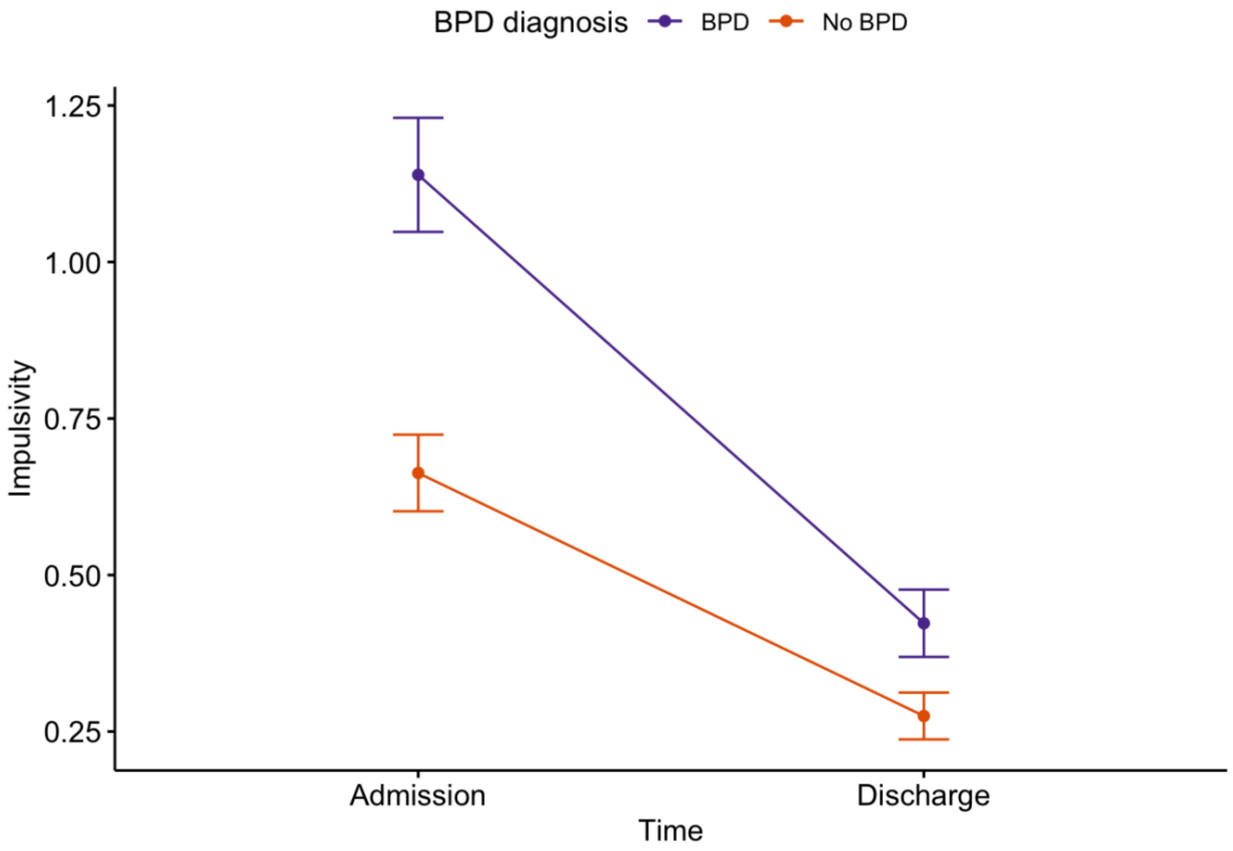

Supplementary Figure 3: Change in impulsive behaviors pre- to post-treatment in naturally cycling females. Naturally cycling females with co-morbid BPD showed greater improvement in impulsive behaviors compared to naturally cycling females without co-morbid BPD.
